# Evaluation of Next Generation Sequencing for Detecting HER2 Copy Number in Breast and Gastric Cancers

**DOI:** 10.1007/s12253-020-00844-w

**Published:** 2020-07-03

**Authors:** Dongfeng Niu, Lei Li, Yang Yu, Wanchun Zang, Zhongwu Li, Lixin Zhou, Ling Jia, Guanhua Rao, Lianju Gao, Gang Cheng, Ke Ji, Dongmei Lin

**Affiliations:** 1 Department of Pathology, Key laboratory of Carcinogenesis and Translational Research (Ministry of Education), Peking University Cancer Hospital & Institute, Beijing, China; 2 Beijing Novogene Bioinformatics Technology Co., Ltd, Beijing, China; 3 Department of Gastrointestinal Surgery, Key laboratory of Carcinogenesis and Translational Research (Ministry of Education), Peking University Cancer Hospital & Institute, Beijing, China

**Keywords:** Next generation sequencing, *HER2* amplification, Breast cancer, Gastric cancer, FISH/IHC

## Abstract

Amplicon-based next generation sequencing (NGS) approaches have been preferentially adopted by the clinical laboratories on the basis of a short turnaround time (TAT) and small DNA input needs. However, little work has been done to assess the amplicon-based NGS methods for copy number variation (CNV) detection in comparison with current standard methods like immunohistochemistry (IHC) and fluorescence in situ hybridization (FISH). The correlation between NGS based CNV detection and the later standard methods has remained unexplored. We developed an amplicon-based panel to detect human epidermal receptor growth factor *(HER2)* amplification in formalin-fixed paraffin-embedded (FFPE) tumor tissue samples from 280 breast cancer and 50 gastric cancer patients. Assessment by IHC and FISH was conducted in parallel, and descriptive statistics were used to assess the concordance. The copy number detected by NGS was correlated with either the average *HER2* copy number (signals/cell) (r = 0.844; *p* < 0.001) or the *HER2*/*CEP17* ratio (r = 0.815; *p* < 0.001). We determined a cut-off value for NGS to categorize *HER2* amplification status by using 151 *HER2* non-amplified FFPE samples. In breast cancer patients, the cut-off value was 2.910, with 95.35%, 98.67% and 97.29% sensitivity, specificity and concordance, respectively. However, this cut-off value displayed low sensitivity in gastric cancer patients (64.71%), and the following macrodissection procedure was not effective for increasing sensitivity (57.14%). Evaluation of *HER2* copy number with NGS in our study was comparable with IHC and FISH in breast cancer patients, but concordance in gastric cancer was only moderate. The greater discordance in gastric cancer may reflect the underlying biological mechanisms, and further study is warranted. NGS-based *HER2* assessment may decrease the equivocal *HER2* determinations in breast cancer patients assessed by FISH/IHC.

**Electronic supplementary material:**

The online version of this article (10.1007/s12253-020-00844-w) contains supplementary material, which is available to authorized users.

## Background

The reliable identification of clinically actionable genomic alteration method is critical for the precision cancer therapy guidance. Next generation sequencing (NGS) has the capability to simultaneously assess multiple genes with a limited biopsy material, thus representing both a cost and tissue-efficient alternative to current single-gene assessment methods [1–3]. Prior studies have shown that NGS enables a reliable detection for copy number variations (CNV) from the same assays used to detect sequence alterations, but less information is available on amplicon-based target sequences [4–7]. CNV calling in the amplicon sequence relies on the calculation of amplicon coverage and suitable normalization. Several factors influence CNV detection, including the number of amplicons per gene, average read dept., and tumor purity within the sample [7]. Thus, an assay and algorithm shall need to be fully validated before being clinically used.

Human epidermal receptor growth factor (*HER2*) amplification or an overexpression occurs in approximately 18–20% of breast cancers, and in nearly 20% of gastric or gastroesophageal junction (GEJ) cancers [8, 9]. Several methods have been recommended for *HER2* amplification assessment, including in situ hybridization (ISH) techniques, which evaluate *HER2* status by measuring the number of *HER2* gene copies, or IHC, which quantifies protein expression [9]. The ASCO/CAP has provided detailed guidelines for conducting and interpreting *HER2* status in a clinical practice. These scoring methods classify cases into “positive”, “negative”, and “equivocal” categories [10]. According to these guidelines, equivocal *HER2* status necessitates additional testing, thus increasing the cost of patient management, and delaying the decision to recommend *HER2*-targeted therapy. The correlation between a copy number called by NGS with an average *HER2* copy number, *HER2*/*CEP17* ratio, or IHC score is not well established. With increasing NGS use in a clinical practice it is increasingly important to validate the amplicon-based detection method against the standard methodologies.

In the current study, we performed and evaluated an amplicon-based NGS assay to assess *HER2* amplification in breast and gastric cancers, by using a custom designed panel and bioinformatics pipeline. We evaluated accuracy and concordance of NGS detection compared with the gold-standard FISH/IHC analysis methodologies.

## Materials and Methods

### Study and Panel Design

For CNV detection, we designed an amplicon-based panel covering 50 genes, which included 13 CNV genes and 6 baseline genes (supplement Tables 1, 2). Briefly, we used cell lines with known amplifications for validating accuracy and had great precision for copy number detection. Then, it was expanded to the FFPE samples from both the breast and gastric cancer patients. We further compared the copy number detected by NGS with the FISH/IHC results from the same sample, and determined a cut-off value of NGS to determine *HER2* status. The study schema is summarized in the supplementary Fig. 1.

### Cell Line CNV Detection

To validate CNV detection, we pooled four cell lines, each bearing single focal gene amplification (*HER2, MET, EGFR* and *FGFR3*) with the matched normal cell lines (GM18511) in several dilution series (40%, 30%, 25%, 20%, 15%, 10%, 5%, 4.5%, 3%). The standard materials list is shown in supplementary Table 3. For orthogonal support, the copy number of the molecular standard materials were also measured by digital PCR using the QuantStudio 3D digital PCR system (Life Technology, CA, USA).

### Patients and Samples

To verify our custom designed 50 gene panel in clinical application, we used FFPE samples from 280 invasive breast cancer patients and 50 gastric cancer patients obtained from Peking University Cancer Hospital. Five FFPE slides, each 5-μm thick, were obtained from breast cancer patient samples along with ten FFPE slides from gastric cancer. Tumors with a high degree of necrosis and < 1000 tumor cells were excluded. More than 80% of the samples we finally selected were samples with tumor purity greater than 20%. This study was approved by the Medical Ethics Committee of Peking University Cancer Hospital, and the investigation was performed in accordance with the Declaration of Helsinki Principles. All patients had signed informed consent for the tissue research, and all of clinical data and samples were deidentified prior to analysis. All the experiments were carried out in accordance with the guideline released by the National Health and Family Planning Commission of the PRC.

### DNA Extraction

Genomic DNA was extracted from unstained FFPE samples using TIANamp FFPE DNA Kit (TIANGEN, Beijing, China), according to manufacturer’s instructions. DNA was quantified using the Qubit dsDNA HS Assay Kit (Life Technology, CA, USA) and the Qubit 2.0 Flurometer (Life Technology, CA, USA) according to recommended protocols. Quality checks were performed by testing 5 ng DNA in 1% agarose gel electrophoresis. Samples in which the main DNA strip in agarose gel electrophoresis less than 600 bp were excluded. The DNA was stored at −20 °C.

### NGS Library Preparation

Sequence libraries were prepared by using library preparation reagents from Life Technology, CA, USA. The amount of DNA input was 15 ng. Libraries were constructed using a custom designed panel (50 hotspot genes). Then, the amplicons by Ion Ampliseq Library Kit 2.0 were barcoded during library generation using the Ion Xpress Barcode Adapters 1–96 Kit. The libraries were purified by AMPure XP beads, quantified using the Ion Library Quantitation Kit, and qualified using Agilent Bioanalyzer 2100. Then the libraries were pooled for sequencing. Multiplex barcoded libraries were enriched by clonal amplification using emulsion PCR on Ion Sphere particles (Ion PI™ Template OT2 200 Kit v3, Life Technology, CA, USA) and loaded on an Ion PI™ Chip. Massively parallel sequencing was carried out on Ion Proton platform using the Ion PI™ Sequencing 200 Kit v3 according to manufacturer’s instructions.

### Sequencing and Data Analysis

Torrent Suite Software (version 4.4.3) was used to perform signal processing, base calling, quality score assignment, and adapter trimming after the sequencing reaction. High quality reads were aligned to human genome 19 reference by tmap4.2.18 software. Quality control and coverage analysis was performed by an in-house analysis pipeline.

### Base Substitution, Short Insertion and Deletion Analysis

TVC (Torrent Variant Caller, version 4.4) was used to call SNV and InDel variants. TVC modules use freebayes to discover candidate variants combined with the hotspots file for detecting gene mutations. Somatic mutations were determined using the following filters: (i) the minimum coverage depth was 100 for SNP and 200 for InDel; (ii) the minimum cutoff of MAF was 0.01 for hotspot variants and 0.05 for others; (iii) detected SNVs and InDels also required at least 25 variant-contained reads to be reported as positive. Those combined minimum coverage, MAF and variant-contained reads to ensure the accuracy of variant calls.

### Copy Number Variation Detection

We used an exome-like approach, rather than the average coverage of exon pull-down regions with read counts per amplicon, for identifying CNV. The coverage of each amplicon was calculated as the number of reads which covered more than one amplicon but mostly aligned to the amplicon. Then, the amplicon-level coverage was divided by the median coverage of each amplicon to normalize or minimize inter-sample variation. The normalized amplicon-level coverage was also corrected by GC content to remove the dependency of coverage across the different GC profiles. Amplicons with a coverage of less than 100 × were excluded from analysis. The copy number ratio of each amplicon was calculated by dividing the GC corrected amplicon-level coverage of tumor samples with that of the matched normal sample or normal pool. In this study, we used a normal pool derived from 14 normal breast cancer patient FFPE samples instead of the matched normal sample for a reference of diploid genome comparison. The copy number ratio was then log-transformed to yield the log2 copy number ratio, which was subsequently used to determine gene amplification status. The gene level fold change was determined as the weighted average of amplicon-level log-copy number ratios, for which the weight of each amplicon was proportional to the number of reads; basically, the reads in the matched normal samples or the normal pool. The final copy number of gene was equal to twice the gene level fold change.

### HER2 IHC and FISH Testing

*HER2* amplification was determined by IHC and the dual probe FISH test. FISH results were reported as average *HER2* copy number and *HER2/CEP17* ratio. All FFPE samples were reviewed by two individual pathologists to determine *HER2* status.

### Statistics

The accuracy (sensitivity and specificity) and precision (repeatability and reproducibility) of NGS was evaluated with standard material result. The correlations of the copy number called by NGS and that determined by digital PCR were studied by using R software. Comparisons of copy numbers which were detected in the three runs, were analyzed using the ANOVA test.

## Results

### Assay Performance of NGS Calls in Standard Materials

**Fig. 1:**
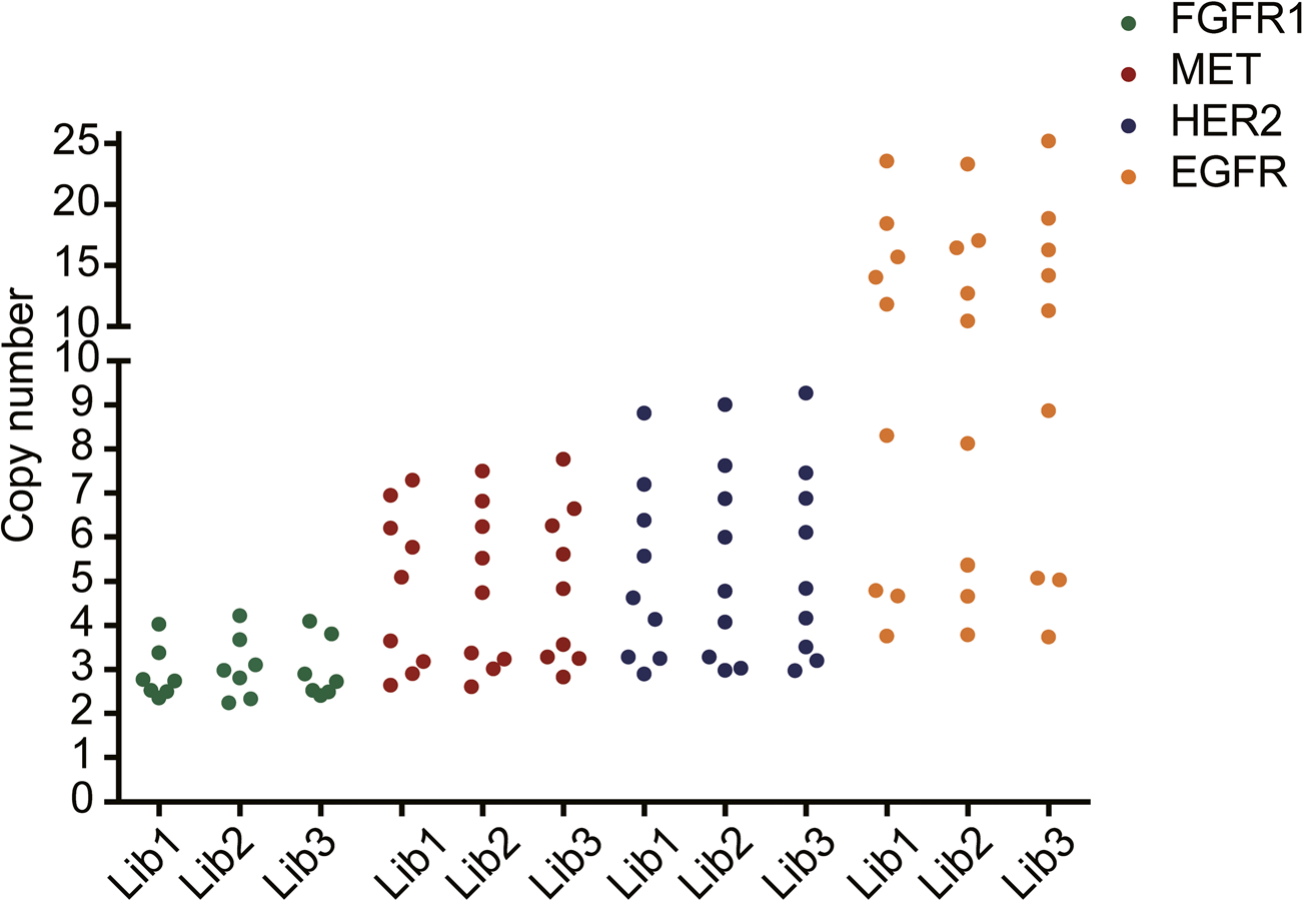
Repeatability of copy number variation detection. NGS-based CNV detections were analyzed using standard materials and replicated libraries

**Fig. 2:**
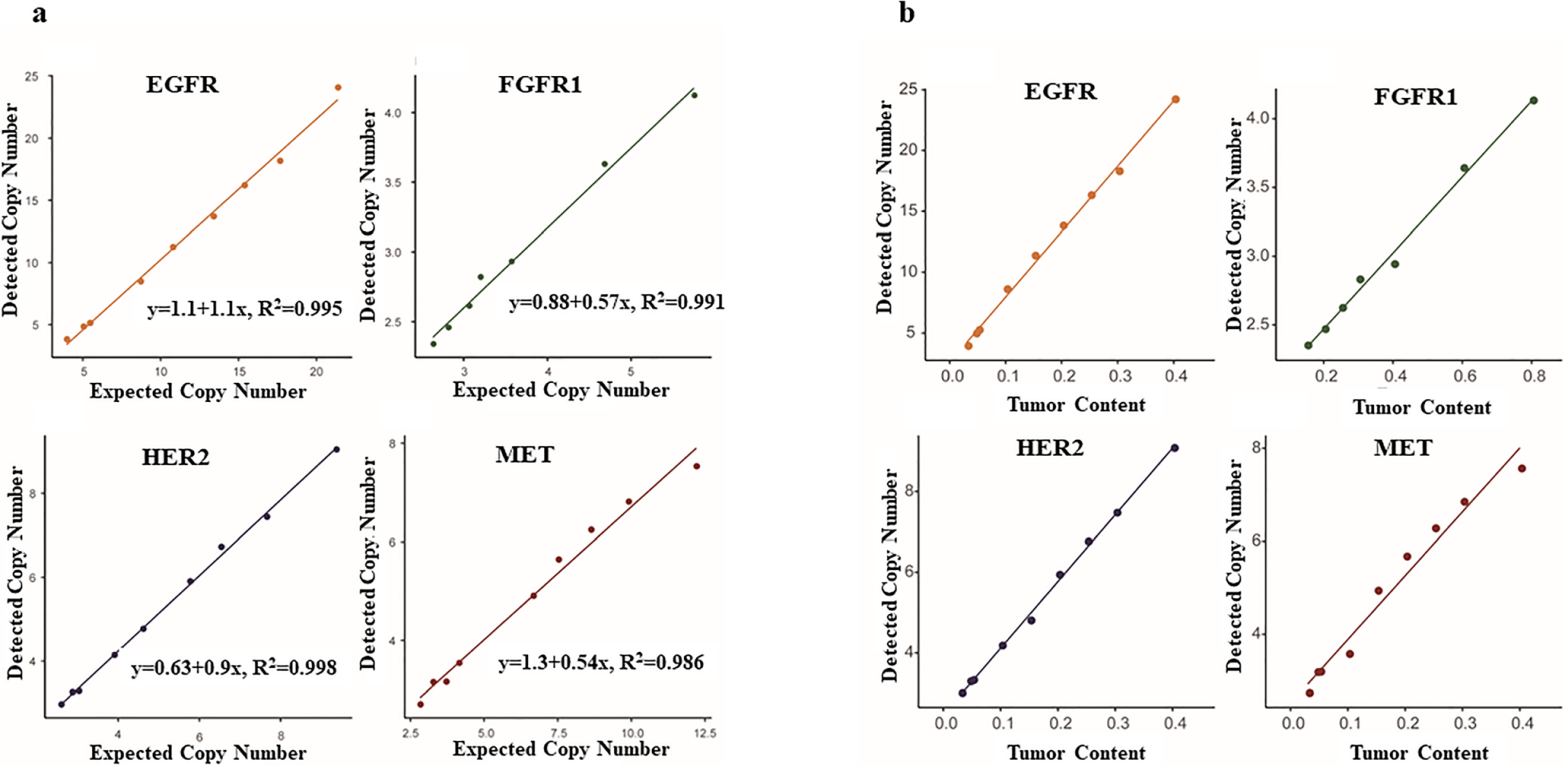
The correlation analysis of CNV detection by preparing libraries. **a** Curve regression analysis between copy number detected by NGS and digital PCR. The copy number detected by digital PCR was used as expected copy number. The copy number detected by NGS was used as detected copy number. **b** The effect of tumor purity on the detection limit for gene amplifications. Four cell lines each bearing single focal gene amplification (*HER2, MET, EGFR* and *FGFR3*) were pooled with their matched normal cell lines in several dilution series. Detection limits were evaluated with dilution series

The NGS assay performance for detecting CNV was analyzed by detecting standard materials. The copy number of standard materials is summarized in Supplementary Table 3. The precision was assessed in inter-assay and intra-assay studies. We first simultaneously ran the two libraries which had been prepared by two different operators (Lib1 and Lib3). Then, Lib1 was done on another run (Lib2), yielding a total of 3 replicates for each sample. Then, it was repeatability evaluated on a per-gene basis among the replicates. Each gene had also a similar copy number estimation in the replicated libraries (Fig. 1). No statistical differences in the copy number could be detected among the three runs (*F-value* = 0.022, *P value* = 0.9783). The coefficient of variation (CV%) for the variation in copy number was <8.58% for inter-assay, and 8.77% for inner-assay, respectively. The total CV% for the three replicates ranged from 0.49% to 7.33% (Supplementary Table 4). A high specimen’s correlation was detected when we compared the copy number for all evaluable genes on the targeted panel with the one generated with digital PCR (Fig. 2a). We also assessed the effect of tumor purity on sensitivity for CNV detection by preparing libraries from tumor DNA diluted with varying amounts of normal DNA, observing that the copy number was linearly related to tumor purity. The level of amplification also greatly impacted the CNV detection using NGS. Genes with a high CNV could be detected with a lower tumor purity than those with a low CNV (Fig. 2b). Subsequent analyses were focused specifically on *HER2* detection.

### Distribution of *HER2* Amplification Status in Breast Cancer

All the 280 FFPE samples from breast cancer patients were tested using FISH and IHC for establishing the *HER2* amplification status. The IHC results showed that 31.4% (88/280) scored as IHC 3+, 185 as IHC 2+ and 7 as IHC 1+. The specimens were then tested by FISH, except for one IHC 2+ sample and one IHC 3+ sample, for which testing had failed. FISH results were 100% consistent with IHC for 1+ and 3+ samples. For 184 IHC 2+ samples, a total of 121 negative samples, 63 positive samples. Two pathologists reviewed these histologic findings, and ranked 143 samples as negative and 41 samples as positive. Overall, this led to 129 positive samples, 151 negative samples.

### Determination and Evaluation of Cut-off to Categorize the *HER2* Status in Breast Cancer Using NGS

**Fig. 3:**
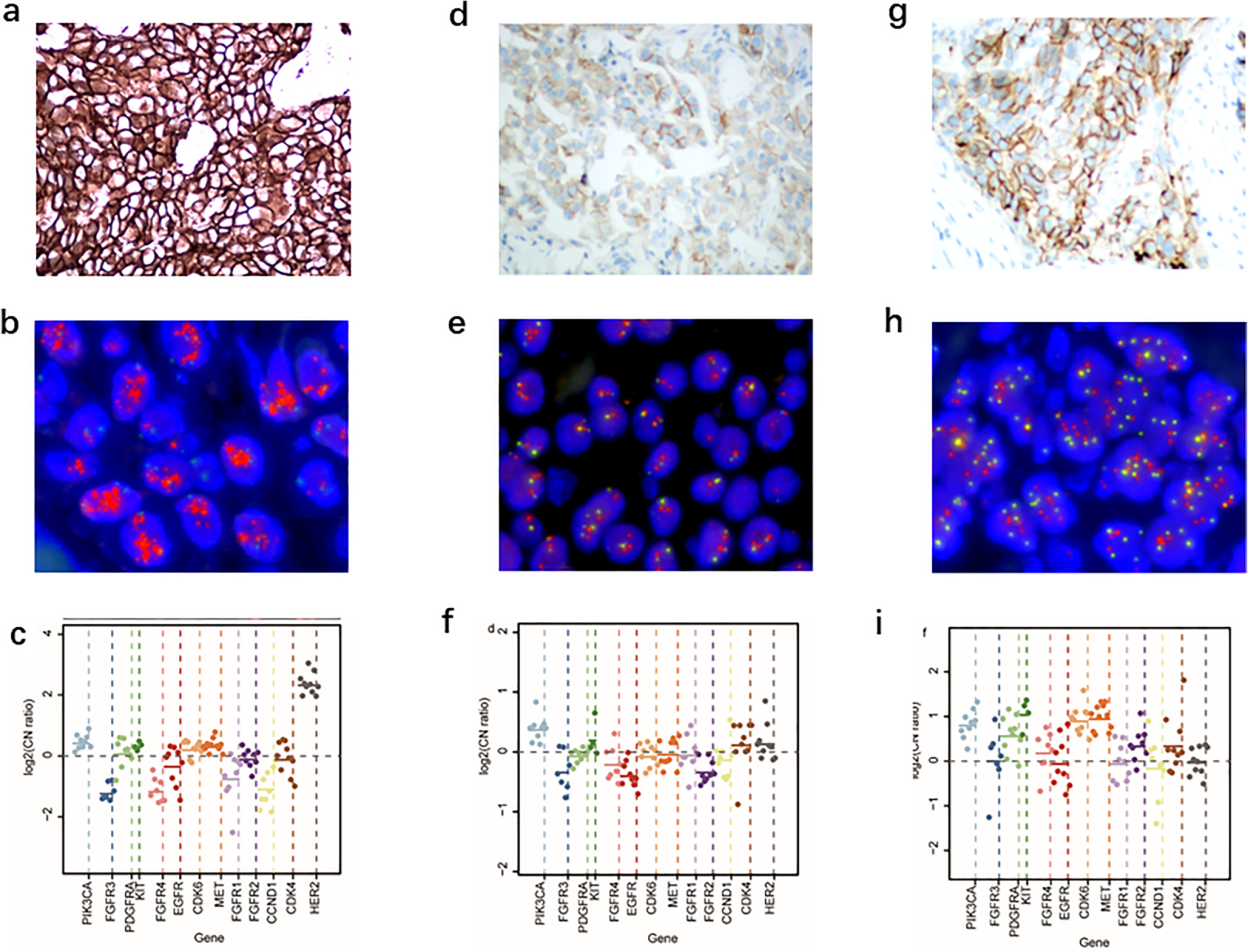
Amplicon-based NGS to detect CNV from breast cancer specimens identified by IHC and FISH. Representative microscopical results of *HER2* amplification (positive, negative and equivocal *HER2* status) were shown by IHC (**a**, **d**, **g**) and dual color FISH (**b**, **e**, **h**). FFPE resection specimens with identified by FISH were further analyzed by NGS (**c**, **f**, **i**). The y-axis shows log2 copy number ratios of each amplicon from each gene

After the validation in the cell lines, we implemented CNV detection in large scale clinical FFPE specimens. The NGS-detected copy number was correlated with the FISH results (Fig. 3). To assess the sensitivity for detecting CNV when compared to the gold-standard method, we evaluated the quantitative correlation between NGS and the average *HER2* copy number or *HER2/CEP17* ratio detected by FISH. We used a total of 255 samples to fit a linear regression model of NGS copy number and the FISH testing results (Supplementary Table 5), excluding those with FISH testing failure (*n* = 3), *HER2* status reviewed by pathologists (*n* = 22). The log10 copy number detected by NGS correlated with either the log10 average *HER2* copy number (signals/cell) (y = 0.044 + 0.73x, r = 0.844; add *p* < 0.001), and log10 *HER2*/*CEP17* ratio (y = 0.26 + 0.74x, r = 0.815; add *p* < 0.001). As NCCN guideline considered average copy number of *HER2* ≥ 6.0 signals/cell as positive, if the average copy number is 6 then the NGS copy number is 4.09 according to this equation. Similarly, if the average copy number is 4, the NGS copy number will be 3.04, and if the *HER2*/*CEP17* ratio is set as 2, the NGS copy number will be 3.03. No false-positive or false-negative samples were found when the copy number detected by NGS was >4.09. This value was hence identified as the confident positive cut-off.

To determine the negative cut-off value, we used 151 *HER2* negative samples to represent the copy number distribution in *HER2* negative samples. The copy number data followed a normal distribution (*P value* of Shapiro-Wilk test = 0.0539). We then used the mean + 3 × MAD (mean absolute deviation) as the negative cut-off value (Supplementary Fig. 2). Two false-positive sample was found when the copy number ranged between 2.91 and 4.09, and five false-negative samples were observed when the copy number was <2.91. Excellent performance was hence found for amplification status when NGS copy number was >4.09 and < 2.91, and the copy number between 2.91 and 4.09 as weak positive status. Overall, we achieved a sensitivity of 95.35% (123/129) and a specificity of 98.67% (149/151) compared with *HER2* status, which was determined by two pathologists who considered both IHC and FISH testing results in breast cancer (Table 1).

**Table 1:** Performance of NGS assay in *HER2* amplification detection in breast cancer patients

Platform		FISH		Total	Sensitivity	Specificity	Concordance
		+	–				
NGS	+	123	2	125	95.35%	98.67%	97.14%
	–	6	149	155			
Total		129	151	280			

### Classification *of HER2* Equivocal Status Samples in Breast Cancer Using NGS

Compared with the HER2 status by IHC score, all 7 IHC 1+ samples were classified as *HER2*-negative by NGS. Among 88 IHC 3+ samples, one (1/88) was identified to be negative by NGS (copy number = 2.71). For 185 IHC 2+ samples, NGS had an 87.80% (36/41) sensitivity and a 98.61% (142/144) specificity compared with *HER2* status determined by two pathologists after considering both IHC and FISH testing.

Seven discrepancies were found, six of which had occurred in the context of a lowered tumor content and *HER2* heterogeneity (Supplementary Fig. 3). The average *HER2* copy number of these samples was 10.6, 5.9, 5.1, 4.1, 13.48 and 7.5, respectively. The *HER2/CEP17* ratio was 3.2, 2.9, 4.7, 2.28, 6.1 and 4.4, respectively. However, the copy number of these samples were < 2.91. All the six specimens displayed high tumor heterogeneity, which was thought to influence NGS accuracy.

### CNV Detection in Gastric Cancer Using NGS

**Fig. 4:**
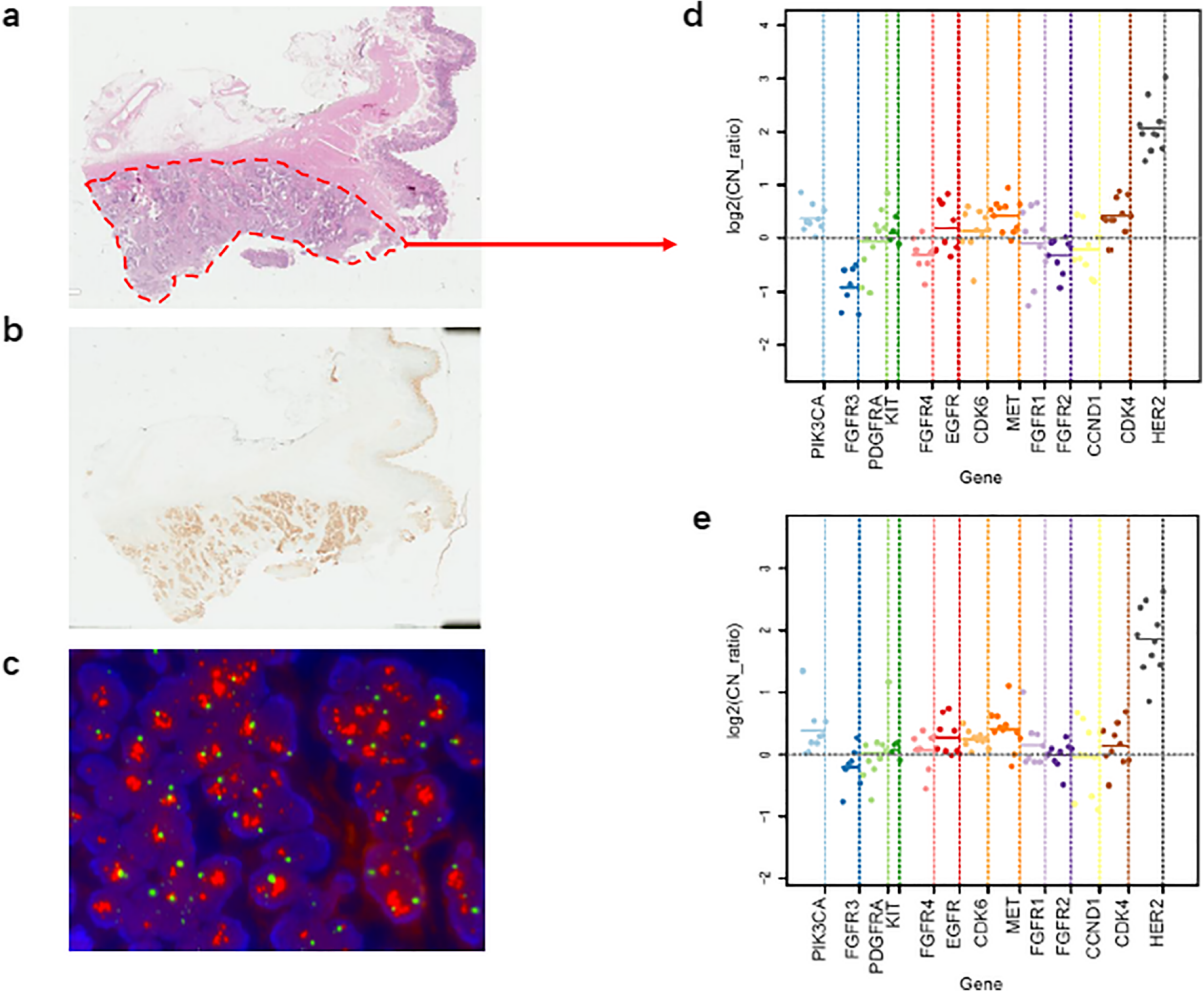
Effect of macrodissection on *HER2* detection by NGS in gastric cancer. **a** Diagram showing macrodissection of FFPE tissue area of HE stained section of gastric cancer. **b** Serial section from the same specimen for HER2 IHC. **c** *HER2* FISH (red signal, *HER2*; green signal, *CEP17*). **d** CNV detection by NGS after macrodissection. **e** CNV detection by NGS before macrodissection

A total number of 50 gastric cancer patients were enrolled in this study. In the initial 39 fully extracted FFPE samples, NGS identified 11 out of 17 *HER2* positive samples, and all the 22 *HER2* negative samples. Although the positive predictive value (PPV) was 100%, NGS categorized all *HER2* negative samples correctly, with a low sensitivity (64.71%, 11/17). Compared with breast cancer, gastric cancer appeared to be more heterogeneous. We hence decided to evaluate whether a macrodissection based IHC result would help to increase the sensitivity. There were 28 *HER2* amplified samples sequenced after macrodissection, and sensitivity was 57.14% (16/28). Although the NGS-detected copy number of most samples increased after macrodissection (Fig. 4), it was not sufficient to improve the accuracy of the NGS assay for *HER2* detection in gastric cancer (Table 2).

**Table 2:** Performance of NGS assay in HER2 amplification detection in gastric cancer

Platform			FISH		Total	Sen	Spe	Con	PPV	NPV
			**+**	**–**						
NGS	Macro dissection	**+**	16	0	16	57.14%	–	57.14%	100%	0.00%
		**–**	12	0	12					
	Full extraction	**+**	11	0	11	64.71%	100%	84.62%	100%	78.57%
		**–**	6	22	28					

### Concurrent Detection of Other Somatic Alterations in the Clinical FFPE Samples

Overall, 118 breast cancer samples with at least one gene mutation detected were include in this study. The most common mutations were *PIK3CA* (109/280), *TP53* (4/280), *AKT1* (3/280), *KRAS* (3/280), *HER2* (2/280), *ALK* (1/280), *EGFR* (1/280), and *RET* (1/280). Among these mutations, annotated based on the OncoKb Knowledge Base [11], *PIK3CA, AKT1* and *HER2* mutations were labelled with a 3A level (Compelling clinical evidence supports the biomarker as being predictive of response to a drug in this indication, but neither biomarker nor drug are standard care). When assigning samples to the level of the most actionable alteration, 40.71% (114/280) patients harbored at least one potentially actionable alteration, which may be a response to a drug, although this is not been defined as standard care so far.

In gastric cancer, *MSH3* was the most common mutation, occurring in 74% (37/50) patients. The frequency of this mutation was higher in gastric cancer without *HER2* amplifications (*HER2*-negative patients: 91%, *HER2*-positive patients: 61%; *p* = 0.036, Chi-square test).

## Discussion

*HER2* amplification has both predictive and prognostic value for breast cancer. Currently, it is regarded as the only biomarker established for selecting specific therapy for patients with advanced gastric cancer [12, 13]. The current gold-standard approach for assessing *HER2* amplification status is based on the IHC and ISH techniques. However, debate continues over the best way to relate *HER2* test results with treatment outcomes. One drawback of the *HER2* test is that the scoring system used to determine *HER2* status is subjective. In this study, we developed an amplicon-based NGS panel to accurately detect clinically relevant copy number alterations. Evaluating *HER2* copy number with NGS in our study yielded comparable results to the gold-standard FISH/IHC analyses in breast cancer patients, achieving 95.35% sensitivity and 98.67% specificity.

The cell line dilution study showed that the detection for amplification is strongly influenced by tumor purity. As shown in Fig. 2b, copy number was reduced in parallel with decrease of tumor purity. For example, the initial copy number was about 4 for FGFR1 at 80% tumor purity, whilst the copy number was approximately 2.45 at 20% tumor purity. Thus, if the initial amplification level was low, it would not be detectable at a low tumor purity. Other studies also shown that samples with poor quality or low DNA content can yield noisy CN plots, thus limiting accurate assessment. The performance was also affected with lower CNVs (6–7 copies) and in samples with poor purity (20–30%) [14]. Thus, determining adequate tumor purity would be mandatory, for an accurate assessment. Furthermore, an alternate method shall be recommended for cases with amplification in combination with low tumor content.

In addition to providing an accurate copy number, we also need to transfer the continuous copy number value into a binary amplification status. In this study, we used known negative samples to determine the negative cut-off value of NGS and established that 2.91 yielded a 95.35% sensitivity and 98.67% specificity when compared to the gold-standard method. To be more confident with NGS CNV detection, we used the established cut-off value for FISH, to estimate a positive cut-off value of NGS according to their correlation. It showed that a copy number using NGS higher than 4.09 corresponded to an average *HER2* copy number higher than 6 signs/cell. Thus, we recommended an NGS copy number between 2.91 and 4.09 to take an additional reflex test. However, it could be foreseen that larger validated samples would reduce this greyscale. In fact, our methods derive a nearly identical cutoff to call *HER2* amplificated by NGS as the large commercial NGS provided Foundation Medicine, Inc. which uses 4 NGS-derived copies to call amplification in *HER2*.

According to updated 2018 ASCO/CAP guideline, concomitant IHC assays are required to arrive at the most accurate *HER2* status designation after *HER2* FISH equivocal results. Currently, our research gave preliminary suggestions, whether dual-probe ISH group 2 to 4 in 2018 ASCO/CAP guideline can be considered for inclusion in the negative. NGS might provide accurate assessment for the *HER2* status designation, and thus reduces the risk of misdiagnosis, and further verification is required.

NGS still has some shortcomings for detection mutations, e.g., CNV detection accuracy was based on accurately assessing coverage depth of genes, which can be biased by high GC content and repetitive regions. A previous study reported that the number of amplicons per gene on the panel may influence performance of CNV detection [7]. The higher number of amplicons per gene would have somehow decreased the variance in CNV assessment, but it would have also restricted the list of assessable genes.

In some cases, metastatic tumors have different molecular alterations from the primary tumors. Even in breast cancer, a discrepancy of *HER2* status between primary tumor and distant metastases has been observed in 7–26% of patients [15]. Regarding gastric cancer, tumor heterogeneity could be precisely identified using ctDNA [16] or planning reflex testing on residual materials or additional tumor blocks. Similar findings have been reported in advanced gastric cancer patients, whose primary tumors were found to be *HER2* negative, but whose circulating tumor cells displayed *HER2* amplification [17].

## Conclusions

Our study demonstrate that an optimized NGS-based test can accurately detect most clinically targetable CNV in a broad spectrum of cancer patients. NGS-based *HER2* assessment may decrease the equivocal *HER2* determinations in breast cancer patients assessed by FISH/IHC. However, due to heterogeneity of gastric cancer tumor tissue, detection of *HER2* amplification by NGS seems still problematic in this malignancy.

## Electronic supplementary material

Supplementary figure 1 The summary of this study. (TIF 710 kb)

High resolution image (PNG 294 kb)

Supplementary figure 2 Distribution of *HER2* copy number from 151 *HER2*-negative FFPE specimens using FISH. A total of 151 *HER2* negative samples were used to determine a cut-off value. The distribution of copy number from 151 samples followed a normal distribution (*P value* of Shapiro-Wilk test = 0.0539). (TIF 306 kb)

High resolution image (PNG 103 kb)

Supplementary figure 3 FISH and NGS results of six discrepancies in breast cancer specimens. a, c, e, g, i and k showed the *HER2* FISH results, whereas b, c, d, g, f, h, j and l showed corresponding NGS detection of *HER2* copy number. The y axis represents the log2 copy number ratio of each amplicon from each gene. (TIF 12266 kb)

High resolution image (PNG 5643 kb)

ESM 4 (DOCX 12 kb)

ESM 5 (DOCX 13 kb)

ESM 6 (DOCX 15 kb)

ESM 7 (DOCX 22 kb)

ESM 8 (DOCX 44.7 kb)

## Abbreviations

NGS: next generation sequencing
CNV: copy number variation
IHC: immunohistochemistry
FISH: fluorescence in situ hybridization
*HER2*: human epidermal receptor growth factor
FFPE: formalin-fixed paraffin-embedded
