# Supplementary figures and images for: Evaluation of Next Generation Sequencing for Detecting HER2 Copy Number in Breast and Gastric Cancers
Source: Pathol Oncol Res. 2020 Jul 3;26(4):2577–85. doi: 10.1007/s12253-020-00844-w (PMC7471150; doi:10.1007/s12253-020-00844-w)

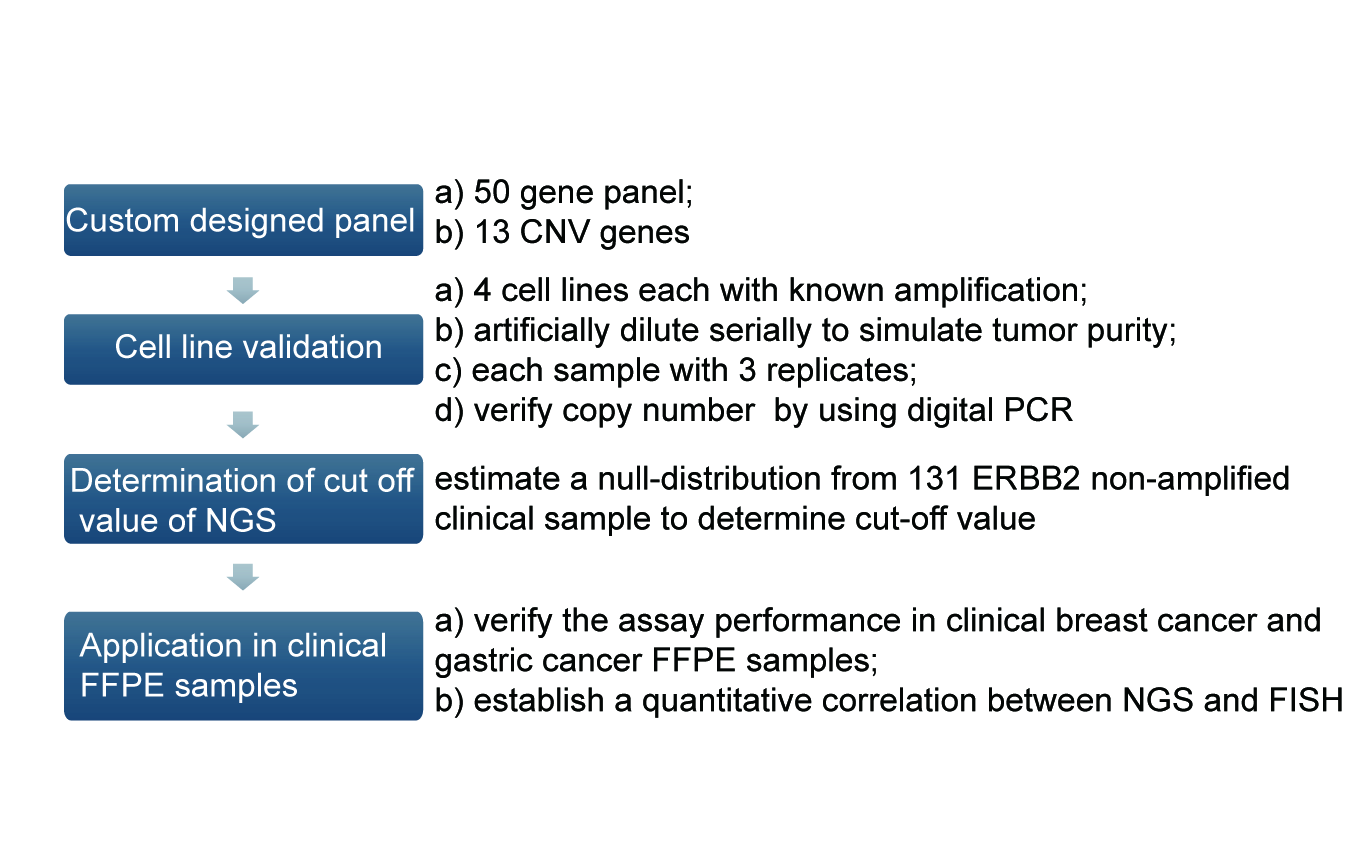

Supplement: Supplementary file 1 — The summary of this study. (TIF 710 kb) [file 12253_2020_844_MOESM1_ESM.tif]

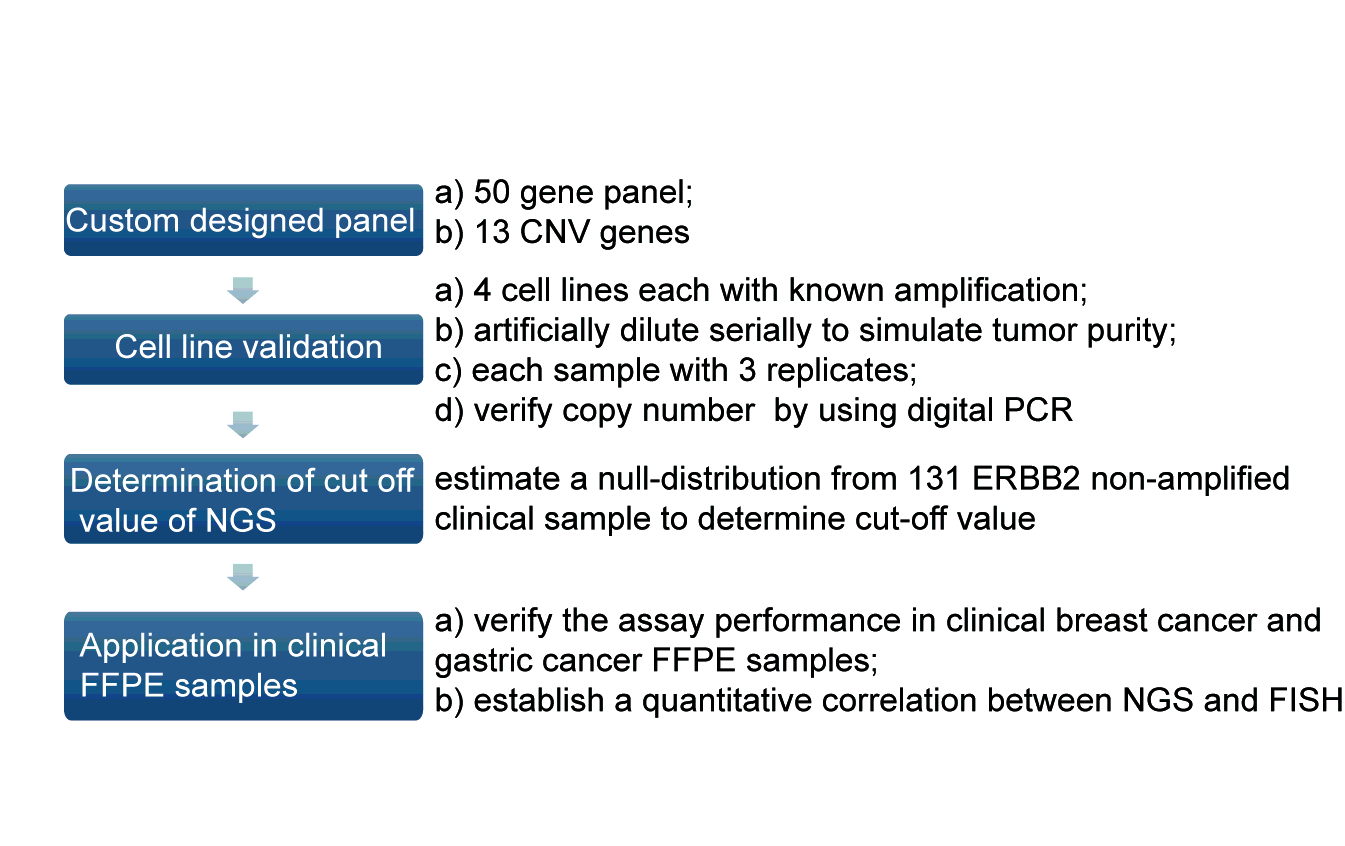

Supplement: Supplementary file 2 — High resolution image (PNG 294 kb) [file 12253_2020_844_Fig5_ESM.png]

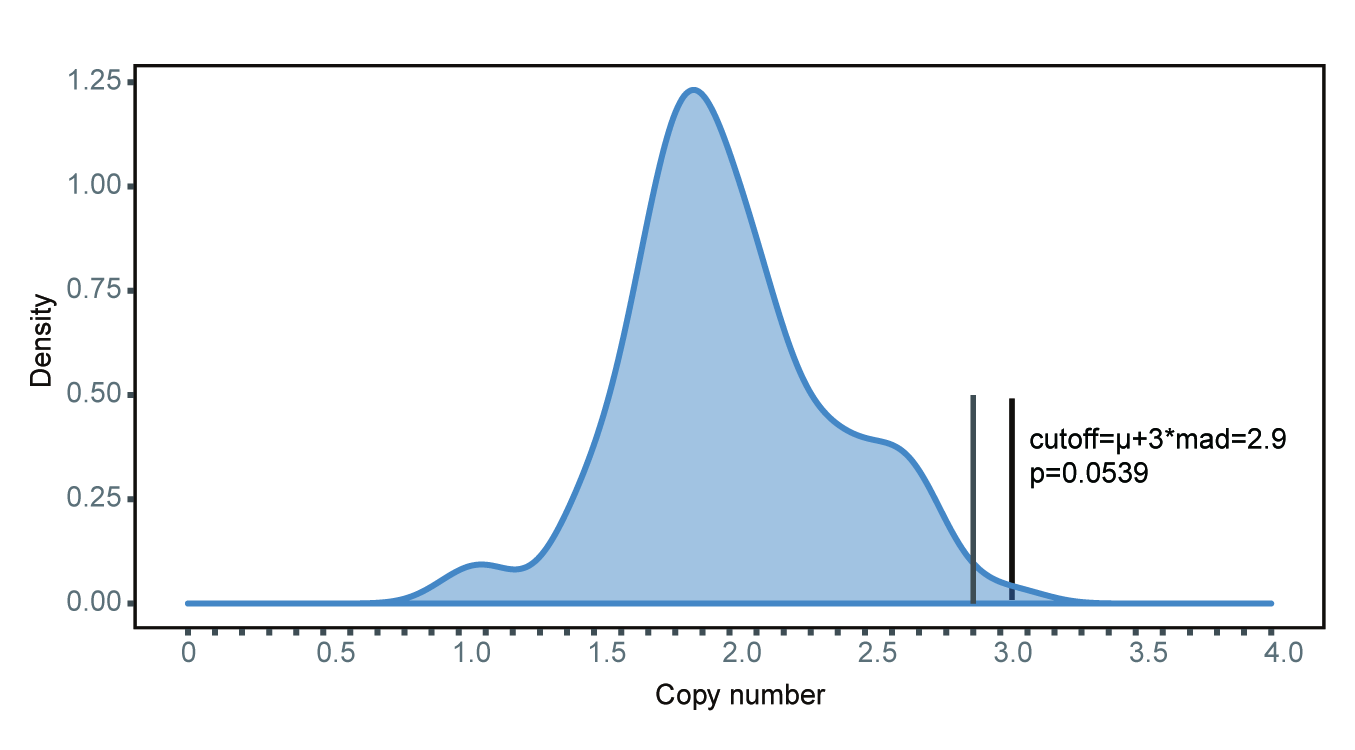

Supplement: Supplementary file 3 — Distribution of HER2 copy number from 151 HER2-negative FFPE specimens using FISH. A total of 151 HER2 negative samples were used to determine a cut-off value. The distribution of copy number from 151 samples followed a normal distribution (P value of Shapiro-Wilk test = 0.0539). (TIF 306 kb) [file 12253_2020_844_MOESM2_ESM.tif]

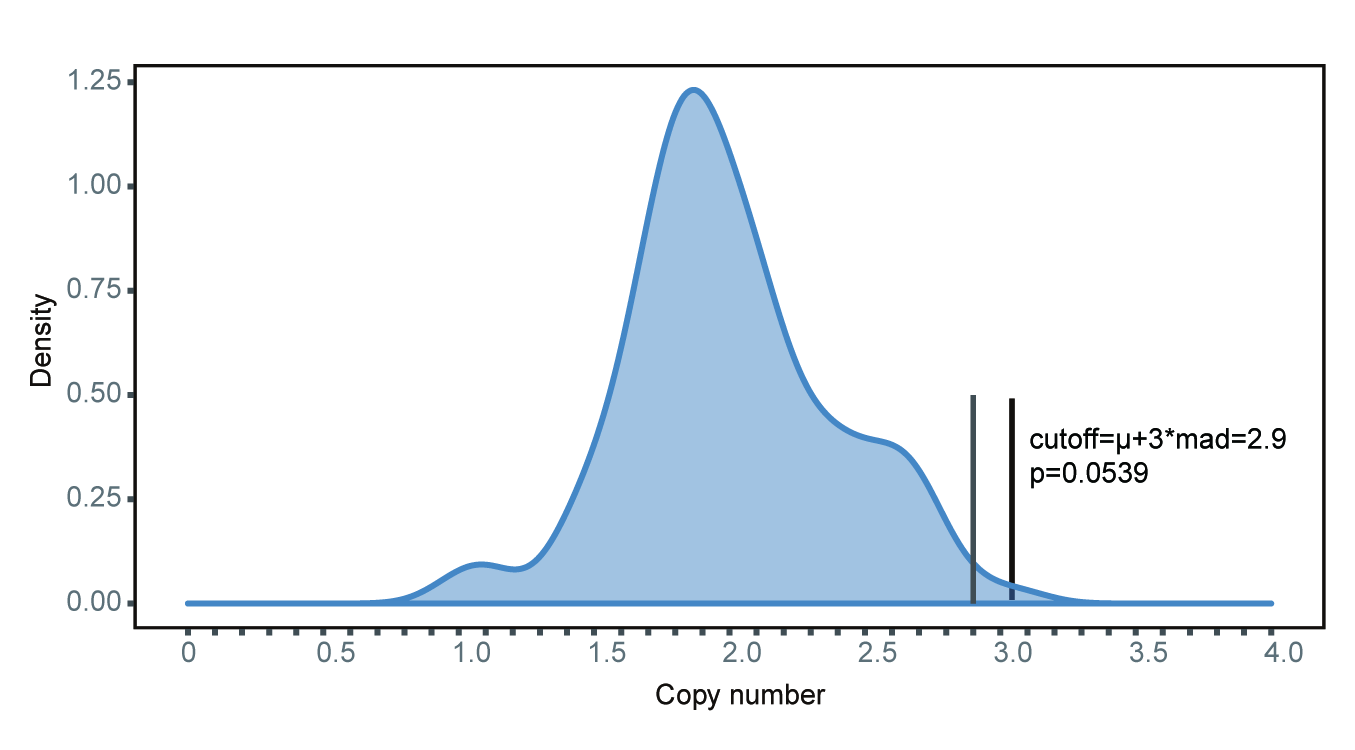

Supplement: Supplementary file 4 — High resolution image (PNG 103 kb) [file 12253_2020_844_Fig6_ESM.png]

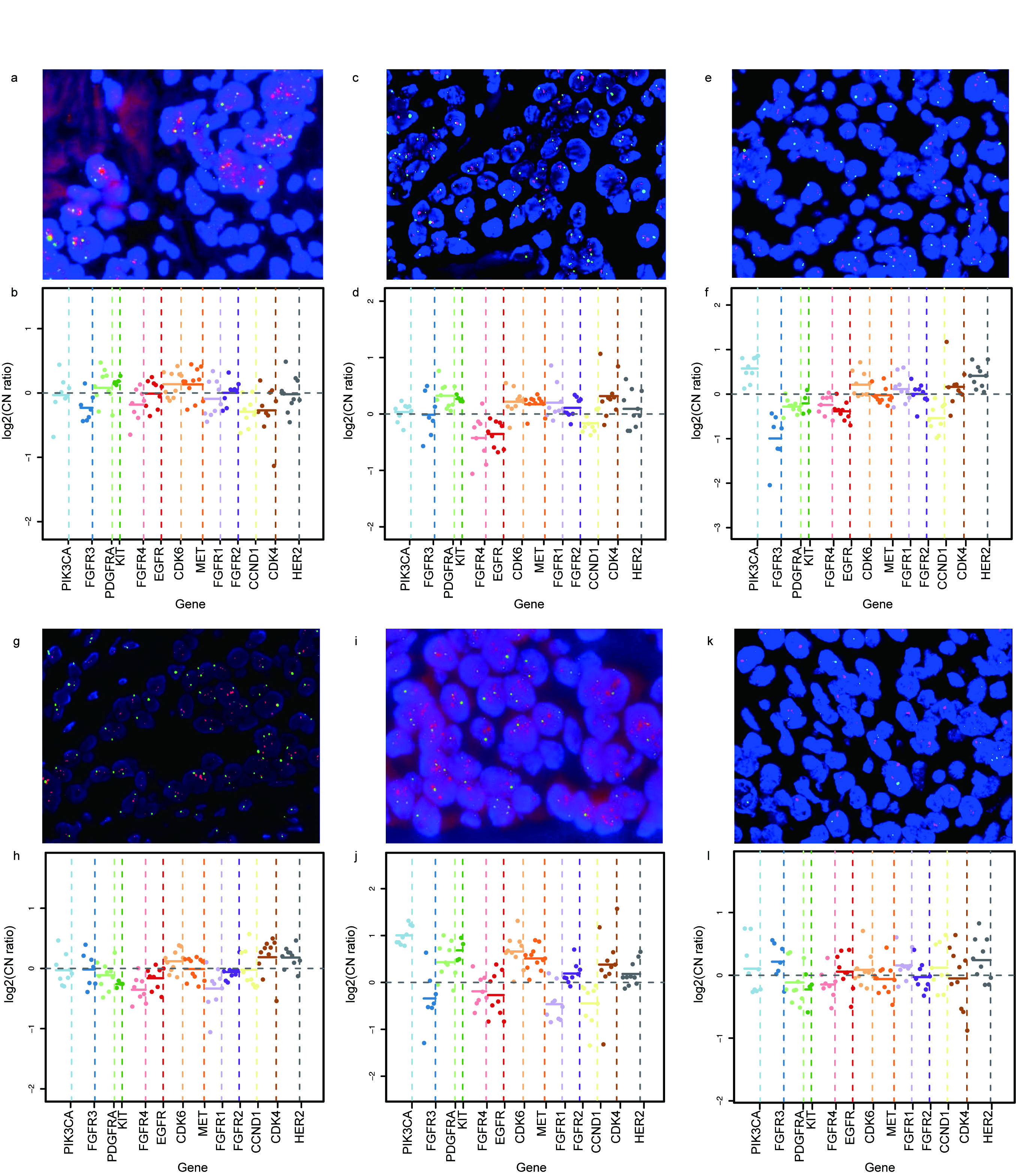

Supplement: Supplementary file 5 — FISH and NGS results of six discrepancies in breast cancer specimens. a, c, e, g, i and k showed the HER2 FISH results, whereas b, c, d, g, f, h, j and l showed corresponding NGS detection of HER2 copy number. The y axis represents the log2 copy number ratio of each amplicon from each gene. (TIF 12266 kb) [file 12253_2020_844_MOESM3_ESM.tif]

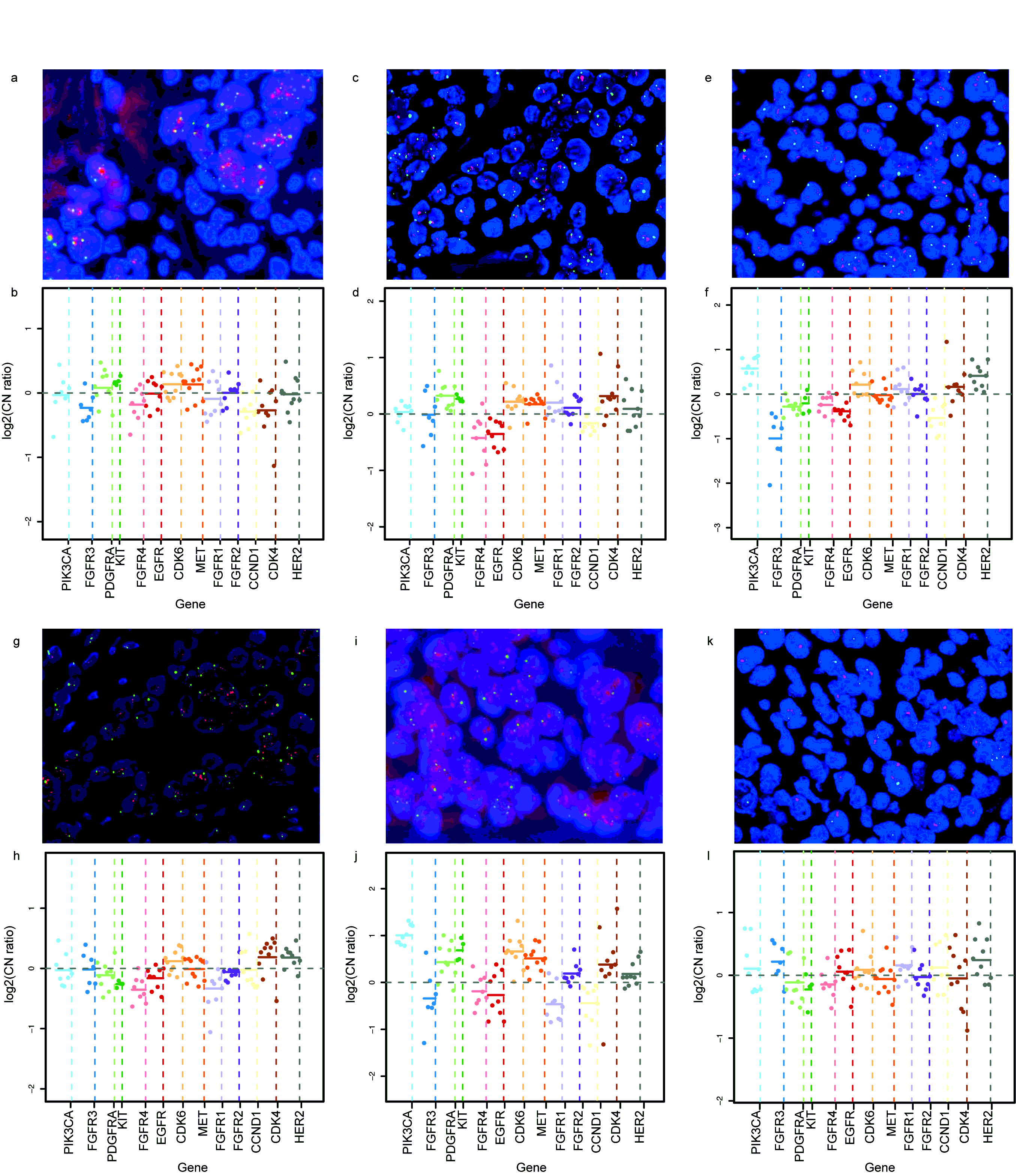

Supplement: Supplementary file 6 — High resolution image (PNG 5643 kb) [file 12253_2020_844_Fig7_ESM.png]
