# Supplementary material for: Evaluation of Next Generation Sequencing for Detecting HER2 Copy Number in Breast and Gastric Cancers
Source: Pathol Oncol Res. 2020 Jul 3;26(4):2577–85. doi: 10.1007/s12253-020-00844-w (PMC7471150; doi:10.1007/s12253-020-00844-w)
Supplement: Supplementary file 7 — (DOCX 12 kb) [file 12253_2020_844_MOESM4_ESM.docx]

Supplement table 1. Gene list of custom designed panel

| Panel | Gene list |
| --- | --- |
| Amplicon based panel (50 genes) | ABCB1 ABCC2 ABCC4 AKT1 ALK APC BIRC2 BRAF BRCA1 C8orf34 CBR3 CCND1 CDA CDK4 CDK6 CYP2B6 CYP2D6 DCUN1D1 DDR2 DHFR DPYD DYNC2H1 EGFR HER2 ERCC1 FCGR3A FGFR1 FGFR2 FGFR3 FGFR4 FOLR3 GGH GSTP1 HRAS KIT KRAS MED12 MEK1 MET MTHFR MTR NF1 NRAS PDGFRA PIK3CA RET SLC19A1 SLC22A16 SMO SOD2 TP53 TSC1 UGT1A1 UMPS XPC XRCC1 |
