# Supplementary material for: Evaluation of Next Generation Sequencing for Detecting HER2 Copy Number in Breast and Gastric Cancers
Source: Pathol Oncol Res. 2020 Jul 3;26(4):2577–85. doi: 10.1007/s12253-020-00844-w (PMC7471150; doi:10.1007/s12253-020-00844-w)
Supplement: Supplementary file 8 — (DOCX 13 kb) [file 12253_2020_844_MOESM5_ESM.docx]

Supplement table 2. CNV gene list and amplicons designed in 50 gene panel

| CNV gene | Number of amplicon | Amplicon size (bp) | Percent on total amplicon regions |
| --- | --- | --- | --- |
| HER2 | 10 | 1165 | 4.82% (1165/24149) |
| MET | 11 | 1326 | 5.49% (1326/24149) |
| EGFR | 10 | 1243 | 5.15% (1243/24149) |
| PIK3CA | 10 | 1161 | 4.81% (1161/24149) |
| FGFR3 | 8 | 935 | 3.87% (935/24149) |
| PDGFRA | 10 | 1223 | 5.06% (1223/24149) |
| FRFG4 | 9 | 1119 | 4.63% (1119/24149) |
| FGFR2 | 10 | 1249 | 5.17% (1249/24149) |
| FGFR1 | 10 | 1223 | 5.06% (1223/24149) |
| CCND1 | 10 | 1177 | 4.87% (1177/24149) |
| CDK4 | 10 | 1240 | 5.13% (1240/24149) |
| CDK6 | 10 | 1229 | 5.09% (1229/24149) |
| Total | 202 | 24149 | - |
