# Supplementary material for: Evaluation of Next Generation Sequencing for Detecting HER2 Copy Number in Breast and Gastric Cancers
Source: Pathol Oncol Res. 2020 Jul 3;26(4):2577–85. doi: 10.1007/s12253-020-00844-w (PMC7471150; doi:10.1007/s12253-020-00844-w)
Supplement: Supplementary file 9 — (DOCX 15 kb) [file 12253_2020_844_MOESM6_ESM.docx]

Supplement table 3. Standard materials list for CNV validation

| Category | Sample ID | Gene | Cell line | Tumor content |
| --- | --- | --- | --- | --- |
| CNV Positive | LISD201 | HER2 | SKBR-3 | 40% |
| CNV Positive | LISD202 | HER2 | SKBR-3 | 30% |
| CNV Positive | LISD203 | HER2 | SKBR-3 | 25% |
| CNV Positive | LISD204 | HER2 | SKBR-3 | 20% |
| CNV Positive | LISD205 | HER2 | SKBR-3 | 15% |
| CNV Positive | LISD206 | HER2 | SKBR-3 | 10% |
| CNV Positive | LISD207 | HER2 | SKBR-3 | 5% |
| CNV Positive | LISD208 | HER2 | SKBR-3 | 4.50% |
| CNV Positive | LISD209 | HER2 | SKBR-3 | 3% |
| CNV Positive | LISD210 | MET | SNU-5 | 40% |
| CNV Positive | LISD211 | MET | SNU-5 | 30% |
| CNV Positive | LISD212 | MET | SNU-5 | 25% |
| CNV Positive | LISD213 | MET | SNU-5 | 20% |
| CNV Positive | LISD214 | MET | SNU-5 | 15% |
| CNV Positive | LISD215 | MET | SNU-5 | 10% |
| CNV Positive | LISD216 | MET | SNU-5 | 5% |
| CNV Positive | LISD217 | MET | SNU-5 | 4.50% |
| CNV Positive | LISD218 | MET | SNU-5 | 3% |
| CNV Positive | LISD219 | EGFR | Hcc827 | 40% |
| CNV Positive | LISD220 | EGFR | Hcc827 | 30% |
| CNV Positive | LISD221 | EGFR | Hcc827 | 25% |
| CNV Positive | LISD222 | EGFR | Hcc827 | 20% |
| CNV Positive | LISD223 | EGFR | Hcc827 | 15% |
| CNV Positive | LISD224 | EGFR | Hcc827 | 10% |
| CNV Positive | LISD225 | EGFR | Hcc827 | 5% |
| CNV Positive | LISD226 | EGFR | Hcc827 | 4.50% |
| CNV Positive | LISD227 | EGFR | Hcc827 | 3% |
| CNV Positive | LISD228 | FGFR1 | H520 | 80% |
| CNV Positive | LISD229 | FGFR1 | H520 | 60% |
| CNV Positive | LISD230 | FGFR1 | H520 | 40% |
| CNV Positive | LISD231 | FGFR1 | H520 | 30% |
| CNV Positive | LISD232 | FGFR1 | H520 | 25% |
| CNV Positive | LISD233 | FGFR1 | H520 | 20% |
| CNV Positive | LISD234 | FGFR1 | H520 | 15% |
