# Supplementary material for: Evaluation of Next Generation Sequencing for Detecting HER2 Copy Number in Breast and Gastric Cancers
Source: Pathol Oncol Res. 2020 Jul 3;26(4):2577–85. doi: 10.1007/s12253-020-00844-w (PMC7471150; doi:10.1007/s12253-020-00844-w)
Supplement: Supplementary file 10 — (DOCX 22 kb) [file 12253_2020_844_MOESM7_ESM.docx]

Supplement table 4. Copy number (CN) detected in standard material with known CNV

| Sample ID | Tumor content | Gene | Digital | NGS | | | Inter-run study | | | Inner study | | | Total | | |
| --- | --- | --- | --- | --- | --- | --- | --- | --- | --- | --- | --- | --- | --- | --- | --- |
|  |  |  | CN | CN | CN | CN | MEAN | SD | CV | MEAN | SD | CV | MEAN | SD | CV |
| LISD201 | 40% | HER2 | 9.37 | 8.82 | 9.01 | 9.27 | 8.91 | 0.14 | 1.52% | 9.04 | 0.32 | 3.57% | 9.03 | 0.23 | 2.53% |
| LISD202 | 30% | HER2 | 7.66 | 7.19 | 7.62 | 7.46 | 7.41 | 0.30 | 4.09% | 7.33 | 0.19 | 2.57% | 7.43 | 0.22 | 2.91% |
| LISD203 | 25% | HER2 | 6.53 | 6.38 | 6.87 | 6.88 | 6.63 | 0.35 | 5.21% | 6.63 | 0.35 | 5.33% | 6.71 | 0.29 | 4.25% |
| LISD204 | 20% | HER2 | 5.77 | 5.57 | 6.00 | 6.11 | 5.78 | 0.30 | 5.26% | 5.84 | 0.38 | 6.57% | 5.89 | 0.29 | 4.86% |
| LISD205 | 15% | HER2 | 4.61 | 4.63 | 4.78 | 4.85 | 4.71 | 0.11 | 2.28% | 4.74 | 0.15 | 3.19% | 4.75 | 0.11 | 2.32% |
| LISD206 | 10% | HER2 | 3.90 | 4.14 | 4.08 | 4.17 | 4.11 | 0.05 | 1.10% | 4.16 | 0.02 | 0.37% | 4.13 | 0.04 | 1.08% |
| LISD207 | 5% | HER2 | 3.02 | 3.28 | 3.03 | 3.51 | 3.16 | 0.18 | 5.64% | 3.40 | 0.16 | 4.74% | 3.28 | 0.24 | 7.33% |
| LISD208 | 4.5% | HER2 | 2.87 | 3.25 | 3.28 | 3.20 | 3.27 | 0.02 | 0.69% | 3.23 | 0.04 | 1.14% | 3.25 | 0.04 | 1.31% |
| LISD209 | 3% | HER2 | 2.59 | 2.90 | 2.99 | 2.98 | 2.94 | 0.06 | 2.07% | 2.94 | 0.06 | 1.88% | 2.95 | 0.05 | 1.61% |
| LISD210 | 40% | MET | 12.20 | 7.30 | 7.50 | 7.77 | 7.40 | 0.14 | 1.93% | 7.53 | 0.33 | 4.41% | 7.52 | 0.24 | 3.14% |
| LISD211 | 30% | MET | 9.90 | 6.95 | 6.81 | 6.64 | 6.88 | 0.09 | 1.38% | 6.80 | 0.22 | 3.18% | 6.80 | 0.15 | 2.26% |
| LISD212 | 25% | MET | 8.62 | 6.20 | 6.24 | 6.26 | 6.22 | 0.03 | 0.43% | 6.23 | 0.04 | 0.68% | 6.23 | 0.03 | 0.49% |
| LISD213 | 20% | MET | 7.52 | 5.77 | 5.52 | 5.61 | 5.64 | 0.17 | 3.08% | 5.69 | 0.11 | 1.96% | 5.63 | 0.12 | 2.21% |
| LISD214 | 15% | MET | 6.67 | 5.10 | 4.75 | 4.84 | 4.93 | 0.25 | 5.03% | 4.97 | 0.19 | 3.73% | 4.90 | 0.18 | 3.72% |
| LISD215 | 10% | MET | 4.14 | 3.65 | 3.38 | 3.57 | 3.51 | 0.19 | 5.52% | 3.61 | 0.06 | 1.57% | 3.53 | 0.14 | 3.99% |
| LISD216 | 5% | MET | 3.71 | 3.19 | 3.02 | 3.25 | 3.10 | 0.12 | 3.88% | 3.22 | 0.05 | 1.49% | 3.15 | 0.12 | 3.89% |
| LISD217 | 4.5% | MET | 3.26 | 2.90 | 3.24 | 3.29 | 3.07 | 0.24 | 7.69% | 3.10 | 0.27 | 8.77% | 3.14 | 0.21 | 6.64% |
| LISD218 | 3% | MET | 2.83 | 2.65 | 2.61 | 2.83 | 2.63 | 0.03 | 0.97% | 2.74 | 0.13 | 4.75% | 2.70 | 0.12 | 4.38% |
| LISD219 | 40% | EGFR | 21.37 | 23.57 | 23.33 | 25.23 | 23.45 | 0.17 | 0.72% | 24.40 | 1.17 | 4.80% | 24.04 | 1.03 | 4.29% |
| LISD220 | 30% | EGFR | 17.65 | 18.44 | 17.05 | 18.88 | 17.75 | 0.98 | 5.53% | 18.66 | 0.31 | 1.64% | 18.12 | 0.95 | 5.25% |
| LISD221 | 25% | EGFR | 15.36 | 15.71 | 16.47 | 16.29 | 16.09 | 0.54 | 3.33% | 16.00 | 0.41 | 2.58% | 16.16 | 0.40 | 2.46% |
| LISD222 | 20% | EGFR | 13.38 | 14.03 | 12.72 | 14.20 | 13.38 | 0.92 | 6.91% | 14.12 | 0.12 | 0.84% | 13.65 | 0.81 | 5.92% |
| LISD223 | 15% | EGFR | 10.77 | 11.82 | 10.47 | 11.31 | 11.14 | 0.96 | 8.58% | 11.56 | 0.36 | 3.13% | 11.20 | 0.68 | 6.10% |
| LISD224 | 10% | EGFR | 8.69 | 8.31 | 8.13 | 8.87 | 8.22 | 0.12 | 1.51% | 8.59 | 0.40 | 4.66% | 8.44 | 0.39 | 4.60% |
| LISD225 | 5% | EGFR | 5.44 | 4.80 | 5.37 | 5.08 | 5.08 | 0.41 | 7.99% | 4.94 | 0.20 | 4.09% | 5.08 | 0.29 | 5.65% |
| LISD226 | 4.5% | EGFR | 5.02 | 4.67 | 4.66 | 5.04 | 4.67 | 0.01 | 0.15% | 4.86 | 0.26 | 5.30% | 4.79 | 0.21 | 4.45% |
| LISD227 | 3% | EGFR | 3.95 | 3.76 | 3.79 | 3.74 | 3.78 | 0.02 | 0.52% | 3.75 | 0.02 | 0.49% | 3.76 | 0.03 | 0.72% |
| LISD228 | 80% | FGFR1 | 5.75 | 4.03 | 4.23 | 4.10 | 4.13 | 0.14 | 3.32% | 4.07 | 0.05 | 1.15% | 4.12 | 0.10 | 2.39% |
| LISD229 | 60% | FGFR1 | 4.68 | 3.39 | 3.68 | 3.81 | 3.53 | 0.21 | 5.88% | 3.60 | 0.30 | 8.37% | 3.63 | 0.22 | 6.01% |
| LISD230 | 40% | FGFR1 | 3.57 | 2.77 | 3.11 | 2.90 | 2.94 | 0.24 | 8.08% | 2.84 | 0.09 | 3.14% | 2.93 | 0.17 | 5.80% |
| LISD231 | 30% | FGFR1 | 3.20 | 2.74 | 2.99 | 2.73 | 2.86 | 0.18 | 6.13% | 2.73 | 0.01 | 0.31% | 2.82 | 0.15 | 5.21% |
| LISD232 | 25% | FGFR1 | 3.06 | 2.53 | 2.81 | 2.49 | 2.67 | 0.20 | 7.48% | 2.51 | 0.02 | 0.90% | 2.61 | 0.17 | 6.62% |
| LISD233 | 20% | FGFR1 | 2.82 | 2.50 | 2.33 | 2.53 | 2.42 | 0.12 | 4.91% | 2.51 | 0.02 | 0.68% | 2.45 | 0.10 | 4.26% |
| LISD234 | 15% | FGFR1 | 2.63 | 2.35 | 2.25 | 2.41 | 2.30 | 0.07 | 3.26% | 2.38 | 0.04 | 1.66% | 2.34 | 0.08 | 3.52% |
