# Supplementary material for: Evaluation of Next Generation Sequencing for Detecting HER2 Copy Number in Breast and Gastric Cancers
Source: Pathol Oncol Res. 2020 Jul 3;26(4):2577–85. doi: 10.1007/s12253-020-00844-w (PMC7471150; doi:10.1007/s12253-020-00844-w)
Supplement: Supplementary file 11 — (DOCX 44.7 kb) [file 12253_2020_844_MOESM8_ESM.docx]

Supplement table 5. CNV identified by NGS and FISH/IHC in FFPE samples from breast cancer patients

| Sample ID | NGS | | FISH/IHC | FISH | | IHC | Concordance |
| --- | --- | --- | --- | --- | --- | --- | --- |
|  | CN | HER2 status | HER2 status | Average HER2 copy number | HER2/CEP17 ratio | IHC score |  |
| 201605209000 | 2.208 | - | - | 2.4 | 0.8 | 1+ | Yes |
| 201605210800 | 1.638 | - | - | 2.1 | 1.03 | 1+ | Yes |
| 201605212600 | 1.516 | - | - | 1.69 | 0.98 | 1+ | Yes |
| 201605215100 | 1.762 | - | - | 2.14 | 1.08 | 1+ | Yes |
| 201605216100 | 2.118 | - | - | 3.3 | 1.7 | 1+ | Yes |
| 201605217300 | 1.042 | - | - | 2.14 | 1.08 | 1+ | Yes |
| 201605219700 | 1.538 | - | - | 2.1 | 1.5 | 1+ | Yes |
| 201605207900 | 2.046 | - | - | 1.68 | 0.95 | 2+ | Yes |
| 201605208000 | 2.09 | - | - | 1.9 | 1.2 | 2+ | Yes |
| 201605208100 | 1.95 | - | - | 1.7 | 1 | 2+ | Yes |
| 201605208200 | 2.65 | - | - | 3.5 | 1.3 | 2+ | Yes |
| 201605208300 | 1.512 | - | - | 1.86 | 0.92 | 2+ | Yes |
| 201605208400 | 2.072 | - | - | 3.7 | 1.6 | 2+ | Yes |
| 201605208500 | 2.556 | - | - | 3.5 | 1.68 | 2+ | Yes |
| 201605208700 | 1.734 | - | - | 1.61 | 0.94 | 2+ | Yes |
| 201605208800 | 2.214 | - | - | 3.5 | 1.5 | 2+ | Yes |
| 201605209100 | 1.118 | - | - | 2.5 | 0.9 | 2+ | Yes |
| 201605209200 | 1.788 | - | - | 2.44 | 1.13 | 2+ | Yes |
| 201605209300 | 1.956 | - | - | 2.06 | 1.23 | 2+ | Yes |
| 201605209400 | 1.988 | - | - | 2.3 | 1.2 | 2+ | Yes |
| 201605209500 | 1.648 | - | - | 1.9 | 0.9 | 2+ | Yes |
| 201605209600 | 1.58 | - | - | 2.9 | 1.3 | 2+ | Yes |
| 201605209700 | 2.104 | - | - | 2.7 | 1.1 | 2+ | Yes |
| 201605209800 | 2.09 | - | - | 2.5 | 1.1 | 2+ | Yes |
| 201605209900 | 2.2 | - | - | 2 | 1.3 | 2+ | Yes |
| 201605210000 | 1.678 | - | - | 2.7 | 1 | 2+ | Yes |
| 201605210100 | 2.746 | - | - | 1.65 | 1.15 | 2+ | Yes |
| 201605210200 | 1.824 | - | - | 1.7 | 1.7 | 2+ | Yes |
| 201605210300 | 2.678 | - | - | 1.9 | 1.5 | 2+ | Yes |
| 201605210400 | 1.688 | - | - | 1.9 | 1.2 | 2+ | Yes |
| 201605210500 | 2.898 | - | - | 1.98 | 1.16 | 2+ | Yes |
| 201605210600 | 2.008 | - | - | 2.82 | 1.28 | 2+ | Yes |
| 201605210700 | 2.114 | - | - | 3.7 | 0.9 | 2+ | Yes |
| 201605210900 | 1.918 | - | - | 2.26 | 1.11 | 2+ | Yes |
| 201605211000 | 1.962 | - | - | 3.3 | 1.4 | 2+ | Yes |
| 201605211200 | 2.13 | - | - | 2.36 | 1.28 | 2+ | Yes |
| 201605211300 | 2.044 | - | - | 3.4 | 1.4 | 2+ | Yes |
| 201605211400 | 2.042 | - | - | 1.56 | 1.39 | 2+ | Yes |
| 201605211600 | 2.138 | - | - | 1.7 | 1.4 | 2+ | Yes |
| 201605211700 | 1.798 | - | - | 1.85 | 1.14 | 2+ | Yes |
| 201605211800 | 2.768 | - | - | 2.34 | 1.36 | 2+ | Yes |
| 201605211900 | 2.134 | - | - | 1.54 | 1.17 | 2+ | Yes |
| 201605212000 | 1.838 | - | - | 2.75 | 1.1 | 2+ | Yes |
| 201605212200 | 1.92 | - | - | 1.95 | 1.16 | 2+ | Yes |
| 201605212300 | 2.42 | - | - | 2.9 | 1.71 | 2+ | Yes |
| 201605212400 | 1.848 | - | - | 2.21 | 1.47 | 2+ | Yes |
| 201605212500 | 1.806 | - | - | 1.7 | 1.2 | 2+ | Yes |
| 201605212700 | 1.922 | - | - | 2.9 | 1.3 | 2+ | Yes |
| 201605212800 | 2.03 | - | - | 3 | 1.2 | 2+ | Yes |
| 201605212900 | 2.71 | - | - | 3.1 | 1.1 | 2+ | Yes |
| 201605213000 | 2.59 | - | - | 3.1 | 1.55 | 2+ | Yes |
| 201605213100 | 2.396 | - | - | 2.1 | 0.91 | 2+ | Yes |
| 201605213200 | 2.036 | - | - | 2.65 | 1.18 | 2+ | Yes |
| 201605213300 | 1.778 | - | - | 1.92 | 1.02 | 2+ | Yes |
| 201605213400 | 1.632 | - | - | 1.82 | 1.1 | 2+ | Yes |
| 201605213600 | 1.454 | - | - | 2.45 | 1.8 | 2+ | Yes |
| 201605213700 | 1.88 | - | - | 3.2 | 1.5 | 2+ | Yes |
| 201605213800 | 2.526 | - | - | 2.7 | 1.42 | 2+ | Yes |
| 201605213900 | 0.98 | - | - | 1.7 | 1 | 2+ | Yes |
| 201605214000 | 1.928 | - | - | 1.9 | 1.1 | 2+ | Yes |
| 201605214100 | 1.834 | - | - | 2.43 | 1.32 | 2+ | Yes |
| 201605214200 | 2.294 | - | - | 1.85 | 1.14 | 2+ | Yes |
| 201605214300 | 1.852 | - | - | 3.05 | 1.33 | 2+ | Yes |
| 201605214500 | 1.88 | - | - | 2.2 | 1.2 | 2+ | Yes |
| 201605214600 | 2.28 | - | - | 1.92 | 1.22 | 2+ | Yes |
| 201605214800 | 1.836 | - | - | 1.52 | 1.06 | 2+ | Yes |
| 201605215200 | 1.734 | - | - | 3.4 | 1.5 | 2+ | Yes |
| 201605215300 | 3.068 | + | - | 2.23 | 1.56 | 2+ | Yes |
| 201605215400 | 2.09 | - | - | 1.8 | 1.1 | 2+ | Yes |
| 201605215500 | 2.424 | - | - | 2.12 | 1.05 | 2+ | Yes |
| 201605215600 | 1.96 | - | - | 2.83 | 1.33 | 2+ | Yes |
| 201605215800 | 2.236 | - | - | 2.35 | 1.62 | 2+ | Yes |
| 201605215900 | 1.91 | - | - | 2.6 | 1.4 | 2+ | Yes |
| 201605216000 | 2.062 | - | - | 2.43 | 1.32 | 2+ | Yes |
| 201605216200 | 2.666 | - | - | 2.67 | 1.27 | 2+ | Yes |
| 201605216300 | 1.468 | - | - | 1.63 | 0.85 | 2+ | Yes |
| 201605216400 | 1.742 | - | - | 2.66 | 1.24 | 2+ | Yes |
| 201605216800 | 1.814 | - | - | 1.92 | 1.34 | 2+ | Yes |
| 201605216900 | 1.654 | - | - | 3.4 | 1 | 2+ | Yes |
| 201605217000 | 1.742 | - | - | 1.85 | 1.11 | 2+ | Yes |
| 201605217100 | 1.87 | - | - | 2.1 | 1.1 | 2+ | Yes |
| 201605217400 | 1.386 | - | - | 2.25 | 0.94 | 2+ | Yes |
| 201605217500 | 1.818 | - | - | 1.7 | 1.1 | 2+ | Yes |
| 201605217600 | 1.698 | - | - | 2.5 | 1.1 | 2+ | Yes |
| 201605217900 | 1.75 | - | - | 2.1 | 1.1 | 2+ | Yes |
| 201605218000 | 1.826 | - | - | 3 | 1.3 | 2+ | Yes |
| 201605218100 | 1.738 | - | - | 1.9 | 1.2 | 2+ | Yes |
| 201605218200 | 1.714 | - | - | 2.75 | 1.1 | 2+ | Yes |
| 201605218300 | 2.024 | - | - | 2.29 | 1.36 | 2+ | Yes |
| 201605218400 | 2.472 | - | - | 2.96 | 1.76 | 2+ | Yes |
| 201605218500 | 1.714 | - | - | 2.01 | 1.19 | 2+ | Yes |
| 201605218600 | 1.844 | - | - | 1.85 | 1.02 | 2+ | Yes |
| 201605218800 | 2.338 | - | - | 1.58 | 1.1 | 2+ | Yes |
| 201605218900 | 1.89 | - | - | 1.96 | 1.12 | 2+ | Yes |
| 201605219000 | 1.83 | - | - | 2.01 | 1.09 | 2+ | Yes |
| 201605219100 | 1.138 | - | - | 1.33 | 1.27 | 2+ | Yes |
| 201605219200 | 1.922 | - | - | 1.62 | 1.16 | 2+ | Yes |
| 201605219300 | 1.664 | - | - | 1.67 | 0.99 | 2+ | Yes |
| 201605219400 | 1.686 | - | - | 1.8 | 1 | 2+ | Yes |
| 201605219600 | 1.824 | - | - | 2.4 | 0.99 | 2+ | Yes |
| 201605219800 | 2.292 | - | - | 2.4 | 1.5 | 2+ | Yes |
| 201605219900 | 1.534 | - | - | 1.95 | 0.91 | 2+ | Yes |
| 201605220000 | 1.698 | - | - | 2.5 | 1.22 | 2+ | Yes |
| 201605220100 | 1.384 | - | - | 1.55 | 0.94 | 2+ | Yes |
| 201605220300 | 1.858 | - | - | 2 | 1.1 | 2+ | Yes |
| 201605220400 | 2.392 | - | - | 2.98 | 1.17 | 2+ | Yes |
| 201605220600 | 1.94 | - | - | 3.5 | 1.1 | 2+ | Yes |
| 201605222900 | 1.608 | - | - | 1.87 | 1.12 | 2+ | Yes |
| 201605200500 | 5.894 | + | + | 5.7 | 2.1 | 2+ | Yes |
| 201605200800 | 10.452 | + | + | 12.5 | 8.9 | 2+ | Yes |
| 201605201100 | 5.552 | + | + | 10.58 | 6.3 | 2+ | Yes |
| 201605201200 | 2.968 | + | + | 12.8 | 4.9 | 2+ | Yes |
| 201605201400 | 3.862 | + | + | 7.7 | 2.2 | 2+ | Yes |
| 201605202300 | 3.296 | + | + | 6.15 | 1.95 | 2+ | Yes |
| 201605202700 | 4.922 | + | + | 10.9 | 8.4 | 2+ | Yes |
| 201605203100 | 2.258 | - | + | 10.6 | 3.2 | 2+ | No |
| 201605203600 | 3.52 | + | + | 7 | 3.7 | 2+ | Yes |
| 201605205300 | 3.758 | + | + | 6.85 | 5.71 | 2+ | Yes |
| 201605205500 | 3.784 | + | + | 6.9 | 4.6 | 2+ | Yes |
| 201605220800 | 2.382 | - | + | 5.9 | 2.9 | 2+ | No |
| 201605221600 | 2.866 | - | + | 5.1 | 4.7 | 2+ | No |
| 201605222400 | 3.04 | + | + | 5.7 | 3.8 | 2+ | Yes |
| 201605222700 | 3.562 | + | + | 7.8 | 3.5 | 2+ | Yes |
| 201605223400 | 4.044 | + | + | 6.3 | 2.33 | 2+ | Yes |
| 201605229500 | 3.06 | + | + | 12.54 | 6.21 | 2+ | Yes |
| 201605229700 | 4.842 | + | + | 8.4 | 2.2 | 2+ | Yes |
| 201605230600 | 3.98 | + | + | 5.1 | 3 | 2+ | Yes |
| 201605232200 | 4.456 | + | + | 3.29 | 2.49 | 2+ | Yes |
| 201605232300 | 7.61 | + | + | 9.4 | 2.3 | 2+ | Yes |
| 201605233300 | 2.59 | - | + | 4.1 | 2.28 | 2+ | No |
| 201605234100 | 4.256 | + | + | 8.69 | 4.12 | 2+ | Yes |
| 201605235200 | 5.292 | + | + | 14.25 | 2.9 | 2+ | Yes |
| 201605225900 | 3.642 | + | + | 3.15 | 2.06 | 2+ | Yes |
| 201605226100 | 4.794 | + | + | 11.44 | 6.57 | 2+ | Yes |
| 201605226200 | 4.984 | + | + | 10.45 | 6.74 | 2+ | Yes |
| 201605226600 | 4.924 | + | + | 5.2 | 3.1 | 2+ | Yes |
| 201605227500 | 3.716 | + | + | 14.35 | 8.24 | 2+ | Yes |
| 201605236300 | 4.876 | + | + | 9.6 | 6.4 | 2+ | Yes |
| 201605237100 | 4.232 | + | + | 6.88 | 3.91 | 2+ | Yes |
| 201605235900 | 3.398 | + | + | 10.36 | 5.05 | 2+ | Yes |
| 201605237600 | 6.192 | + | + | 11.36 | 6.11 | 2+ | Yes |
| 201605237700 | 4.898 | + | + | 11.36 | 6.11 | 2+ | Yes |
| 201605240400 | 6.344 | + | + | 8.5 | 7.1 | 2+ | Yes |
| 201605239200 | 2.59 | - | + | 13.48 | 6.1 | 2+ | No |
| 201605239400 | 3.74 | + | + | 6.6 | 3.8 | 2+ | Yes |
| 201605239500 | 2.946 | + | + | 6.6 | 3.3 | 2+ | Yes |
| 201605238300 | 6.35 | + | + | 9.6 | 6.4 | 2+ | Yes |
| 201605238400  201605201000  201605201500  201605201600  201605201700  201605201800  201605201900  201605202000  201605202100  201605202200  201605203800  201605203900  201605204000  201605204200  201605204300  201605204400  201605204500  201605204600  201605204700  201605231900  201605235600 | 5.4  2.632  2.468  2.590  2.562  2.340  3.010  2.172  1.974  1.898  2.452  1.958  1.776  2.566  2.706  1.676  1.898  2.166  2.250  2.338  2.436 | +  -  -  -  -  -  -  -  -  -  -  -  -  -  -  -  -  -  -  -  - | +  -  -  -  -  -  -  -  -  -  -  -  -  -  -  -  -  -  -  -  - | 14.39  7.00  4.50  4.80  4.40  4.70  4.30  4.65  4.60  4.60  4.40  5.00  4.60  4.60  4.65  4.80  4.90  5.70  4.80  7.20  6.50 | 5.71  1.40  1.70  1.60  1.50  1.40  1.30  1.16  1.40  1.40  1.30  1.90  1.35  1.35  1.41  1.10  1.30  1.80  1.40  2.06  1.90 | 2+  2+  2+  2+  2+  2+  2+  2+  2+  2+  2+  2+  2+  2+  2+  2+  2+  2+  2+  2+  2+ | Yes  Yes  Yes  Yes  Yes  Yes  No  Yes  Yes  Yes  Yes  Yes  Yes  Yes  Yes  Yes  Yes  Yes  Yes  Yes  Yes |
| 201605200100 | 6.852 | + | + | 15.23 | 9.4 | 3+ | Yes |
| 201605200200 | 9.342 | + | + | 20.5 | 12.8 | 3+ | Yes |
| 201605200400 | 13.726 | + | + | 10.58 | 5.69 | 3+ | Yes |
| 201605201300 | 5.146 | + | + | 15.25 | 8.07 | 3+ | Yes |
| 201605202900 | 7.236 | + | + | 12.63 | 5.31 | 3+ | Yes |
| 201605203000 | 8.304 | + | + | 15.23 | 7.05 | 3+ | Yes |
| 201605203200 | 4.908 | + | + | 12.98 | 7.13 | 3+ | Yes |
| 201605203300 | 5.758 | + | + | 10.5 | 4.4 | 3+ | Yes |
| 201605203400 | 12.882 | + | + | 8.2 | 3 | 3+ | Yes |
| 201605203500 | 3.278 | + | + | 24.38 | 14.51 | 3+ | Yes |
| 201605203700 | 17.022 | + | + | 30.25 | 19.39 | 3+ | Yes |
| 201605205000 | 12.494 | + | + | 16.5 | 3.7 | 3+ | Yes |
| 201605205200 | 10.146 | + | + | 35.18 | 13.58 | 3+ | Yes |
| 201605206500 | 23.178 | + | + | 30.25 | 16.26 | 3+ | Yes |
| 201605206600 | 13.214 | + | + | 25.36 | 13.42 | 3+ | Yes |
| 201605206700 | 7.262 | + | + | 17.5 | 5 | 3+ | Yes |
| 201605206800 | 6.184 | + | + | 10.69 | 5.78 | 3+ | Yes |
| 201605206900 | 11.212 | + | + | 26.21 | 14.64 | 3+ | Yes |
| 201605207100 | 8.236 | + | + | 12 | 6 | 3+ | Yes |
| 201605220900 | 10.308 | + | + | 10.36 | 5.21 | 3+ | Yes |
| 201605221200 | 3.184 | + | + | 9.5 | 5.3 | 3+ | Yes |
| 201605221500 | 9.402 | + | + | 18.15 | 8.21 | 3+ | Yes |
| 201605221700 | 4.136 | + | + | 8 | 3.2 | 3+ | Yes |
| 201605221800 | 7.742 | + | + | 11 | 2.2 | 3+ | Yes |
| 201605221900 | 5.52 | + | + | 6.4 | 3.2 | 3+ | Yes |
| 201605222000 | 7.238 | + | + | 6.39 | 3.12 | 3+ | Yes |
| 201605222600 | 10.264 | + | + | 6.35 | 2.36 | 3+ | Yes |
| 201605222800 | 5.346 | + | + | 7.2 | 3.3 | 3+ | Yes |
| 201605223500 | 23.254 | + | + | 15.26 | 6.39 | 3+ | Yes |
| 201605223900 | 4.304 | + | + | 12.9 | 6.6 | 3+ | Yes |
| 201605224000 | 13.89 | + | + | 17.25 | 10.26 | 3+ | Yes |
| 201605224100 | 5.14 | + | + | 4.8 | 2.6 | 3+ | Yes |
| 201605224200 | 2.994 | + | + | 7.4 | 1.85 | 3+ | Yes |
| 201605224400 | 7.456 | + | + | 8.3 | 4.6 | 3+ | Yes |
| 201605224600 | 6.812 | + | + | 6.7 | 4.8 | 3+ | Yes |
| 201605224700 | 17.106 | + | + | 21.26 | 11.29 | 3+ | Yes |
| 201605224900 | 3.518 | + | + | 8.9 | 4.2 | 3+ | Yes |
| 201605225100 | 9.882 | + | + | 16.74 | 7.18 | 3+ | Yes |
| 201605225200 | 3.63 | + | + | 8.4 | 6.5 | 3+ | Yes |
| 201605228800 | 46.608 | + | + | 8.6 | 4.5 | 3+ | Yes |
| 201605229100 | 20.958 | + | + | 20.39 | 9.26 | 3+ | Yes |
| 201605229300 | 10.274 | + | + | 10.3 | 6.1 | 3+ | Yes |
| 201605229400 | 10.092 | + | + | 15.36 | 8.17 | 3+ | Yes |
| 201605229600 | 8.696 | + | + | 15.28 | 7.36 | 3+ | Yes |
| 201605229800 | 10.58 | + | + | 16.58 | 9.27 | 3+ | Yes |
| 201605229900 | 5.56 | + | + | 18.39 | 10.13 | 3+ | Yes |
| 201605230000 | 10.402 | + | + | 20.69 | 12.47 | 3+ | Yes |
| 201605230100 | 8.742 | + | + | 7.24 | 3.7 | 3+ | Yes |
| 201605230200 | 14.238 | + | + | 19.37 | 9.38 | 3+ | Yes |
| 201605230400 | 12.546 | + | + | 17.68 | 9.89 | 3+ | Yes |
| 201605230500 | 5.272 | + | + | 8.42 | 3.8 | 3+ | Yes |
| 201605231100 | 4.254 | + | + | 9.3 | 4.89 | 3+ | Yes |
| 201605231300 | 4.93 | + | + | 8.39 | 2.95 | 3+ | Yes |
| 201605231700 | 9.224 | + | + | 17.39 | 9.68 | 3+ | Yes |
| 201605232000 | 6.278 | + | + | 16.38 | 8.67 | 3+ | Yes |
| 201605232100 | 14.278 | + | + | 15.25 | 7.06 | 3+ | Yes |
| 201605232500 | 16.206 | + | + | 18.36 | 8.39 | 3+ | Yes |
| 201605232800 | 3.126 | + | + | 9.26 | 4.68 | 3+ | Yes |
| 201605232900 | 11.886 | + | + | 12.65 | 6.59 | 3+ | Yes |
| 201605233000 | 7.67 | + | + | 9.6 | 4.6 | 3+ | Yes |
| 201605233600 | 9.004 | + | + | 6.4 | 2.5 | 3+ | Yes |
| 201605233400 | 6.354 | + | + | 12.1 | 5 | 3+ | Yes |
| 201605233800 | 9.624 | + | + | 10.4 | 4.3 | 3+ | Yes |
| 201605234000 | 11.116 | + | + | 8.6 | 4.8 | 3+ | Yes |
| 201605234700 | 8.978 | + | + | 30.2 | 2.2 | 3+ | Yes |
| 201605234800 | 6.396 | + | + | 18.26 | 10.22 | 3+ | Yes |
| 201605234900 | 3.132 | + | + | 21.36 | 11.55 | 3+ | Yes |
| 201605235000 | 2.71 | - | + | 7.5 | 4.4 | 3+ | No |
| 201605235400 | 6.824 | + | + | 8.7 | 4.1 | 3+ | Yes |
| 201605235500 | 12.302 | + | + | 2.8 | 2.15 | 3+ | Yes |
| 201605225600 | 12.514 | + | + | 8.2 | 6.3 | 3+ | Yes |
| 201605225700 | 6.102 | + | + | 12.29 | 6.11 | 3+ | Yes |
| 201605225800 | 6.432 | + | + | 7.9 | 5.6 | 3+ | Yes |
| 201605226700 | 17.022 | + | + | 18.35 | 1.36 | 3+ | Yes |
| 201605231800 | 13.75 | + | + | 21.66 | 12.14 | 3+ | Yes |
| 201605236100 | 8.572 | + | + | 25.5 | 17 | 3+ | Yes |
| 201605236500 | 11.75 | + | + | 12.5 | 7.8 | 3+ | Yes |
| 201605236600 | 10.32 | + | + | 11.5 | 3.7 | 3+ | Yes |
| 201605236900 | 3.956 | + | + | 10.5 | 3.2 | 3+ | Yes |
| 201605236000 | 4.638 | + | + | 11.5 | 2.1 | 3+ | Yes |
| 201605237800 | 22.838 | + | + | 19.5 | 12.2 | 3+ | Yes |
| 201605240500 | 5.848 | + | + | 10.5 | 3.2 | 3+ | Yes |
| 201605238700 | 16.138 | + | + | 16.5 | 10.3 | 3+ | Yes |
| 201605238900 | 11.124 | + | + | 12.55 | 6.64 | 3+ | Yes |
| 201605239700 | 8.64 | + | + | 15.32 | 4.44 | 3+ | Yes |
| 201605238000 | 4.95 | + | + | 9.2 | 3.7 | 3+ | Yes |
| 201605238200 | 11.724 | + | + | 10.5 | 6.2 | 3+ | Yes |
